# Antiretroviral drug use by individuals living with HIV/AIDS and compliance with the Clinical Protocol and Therapy Guidelines

**DOI:** 10.31744/einstein_journal/2020AO4995

**Published:** 2020-02-13

**Authors:** Lucas Eduardo Fedaracz Brojan, Leticia Mara Marca, Frederico Alves Dias, Yanna Dantas Rattmann

**Affiliations:** 1 Universidade Federal do Paraná CuritibaPR Brazil Universidade Federal do Paraná, Curitiba, PR, Brazil.

**Keywords:** Anti-retroviral agents, Prescriptions, Guidelines, Combined modality therapy, Observational study, Clinical protocols

## Abstract

**Objective:**

To describe antiretroviral treatment regimens prescribed and their compliance with the Clinical Protocol and Therapy Guidelines of the Ministry of Health for the management of HIV infection.

**Methods:**

Observational and descriptive study. Secondary data of the state of Paraná (Brazil) on drugs, treatment regimens, lines of treatment and number of individuals on treatment, from January to June 2018, were accessed at the Antiretroviral Agents Logistic Control System. Combinations of antiretroviral drugs (treatment regimens) were compared according to the current Clinical Protocol and Therapy Guidelines and non-compliances were classified and quantified.

**Results:**

In Paraná, 35,127 individuals with HIV were treated with 253 different treatment regimens. Of the prescribed regimens, 19.1% were first-line, 27.4% second-line and 48.5% third-line. Among non-compliances, the most prevalent were absence of association of protease inhibitors and ritonavir (42.8%), low efficacy triple therapy (36.9%), double therapy (26.1%), monotherapy (20.3%), and triple therapy of nucleoside analog reverse transcriptase inhibitors (17.1%).

**Conclusion:**

Most individuals receiving HIV treatment in the state of Paraná are on treatment regimens established in the current Clinical Protocol and Therapy Guidelines, which contributes to successful therapy. However, associations not provided by the current Clinical Protocol and Therapy Guidelines were identified in the initial treatment lines, which could lead to ineffectiveness, virologic failure and viral resistance.

## INTRODUCTION

The infection caused by the human immunodeficiency virus (HIV) is a major issue for community health in Brazil and worldwide, due to morbidity and mortality, and the consequent impact on public health policies.^( 1 )^

In 1996, federal law 9.313 was enacted and guaranteed access to antiretroviral therapy through the Unified Health System (SUS - *Sistema Único de Saúde* ).^( 2 )^ Studies have shown that this HIV infection control-oriented public policy contributed significantly to the reduction in mortality and hospitalizations due to HIV/AIDS in Brazil.^( 3 , 4 )^

Currently, the SUS has 21 drugs available for controlling HIV infection. They are distributed into six distinctive pharmacological classes: nucleoside analog reverse-transcriptase inhibitors (NRTI) and non-nucleoside reverse-transcriptase inhibitors (NNRTI), which prevent replication of viral RNA inside TCD4+ cells; protease inhibitors (PI), which block the enzyme that breaks viral proteins synthetized in host cells; integrase inhibitors (INI), which inhibit the enzyme that integrates viral RNA to the DNA of host cells; fusion inhibitors (FI), which prevent the fusion of the viral membrane to the human cell membrane; and CCR5 inhibitors, which inhibit membrane proteins that bind to the HIV and prevent the infection in host cells.^( 5 )^

The Clinical Protocol and Therapy Guidelines (PCDT - *Protocolo Clínico e Diretrizes Terapêuticas* ) of the Ministry of Health, aimed to manage HIV infection, guides the choice of prescriptions through treatment regimens comprised by combinations of more than one antiretroviral drug, organized in different lines of therapy. Treatment success attained by treatment regimens provides reduction in the number of viral copies, increase in the number of TCD4+ lymphocytes and consequent restoration of immunity.^( 6 )^

First line therapy consists in the treatment regimen prescribed right after diagnosis. If viral suppression and restoration of immunity is unsuccessful, second line therapy should be prescribed, and so on and so forth. Treatment failures can occur due to adverse reactions to drugs, less effective regimens, poor compliance, and transmitted viral resistance. Under these circumstances, antiretroviral regimens are changed, and may in more critical scenarios result in customized regimens, guided by genotyping, not anticipated by the PCDT.^( 6 , 7 )^

It therefore becomes important to identify drugs being used and their associations, and compliance to PCDT, in order to contribute to the rational use of antiretrovirals and control of the infection.

## OBJECTIVE

To describe antiretroviral drugs and regimens prescribed and their compliance with the Clinical Protocol and Therapy Guidelines.

## METHODS

Observational and descriptive study. Secondary data, referring to the period between January and June 2018 were accessed on the Antiretroviral Drug Logistic Control System (SICLOM - *Sistema de Controle Logístico de Medicamentos Antirretrovirais* ) through the Paraná Medication Center (CEMEPAR - *Centro de Medicamentos do Paraná* ), of the Paraná State Department of Health (SESA-PR - *Secretaria de Estado da Saúde do Paraná* ).

Data on antiretroviral dispensing in the state of Paraná, stratified by treatment regimens (combination of different antiretrovirals) dispensed, per lines of treatment regimens, and per number of users were attained.

Information was computed and then classified according to compliance or non-compliance with the Ministry of Health PCDT to manage HIV infection (PCDT 2017). Non-compliances were identified and stratified into the following categories: monotherapy, double therapy not allowed in the PCDT in effect, low-efficiency triple therapy, non-association PI + ritonavir, double PI, three NRTI, therapeutic duplicity, and possible genotyping-guided treatment regimens.

The study was approved by the Ethics Committee of *Universidade Federal do Paraná* and of the Paraná State Health Department (SESA-PR), under CAAE: 82936318.3.3001.5225 and opinion 2.674.606.

## RESULTS

In the period from January to June 2018, 235 different treatment regimens prescribed to 35,127 individuals living with HIV/AIDS in Paraná were identified. Of the 21 antiretroviral drugs standardized by SUS, 18 were prescribed at least once ( Table 1 ).

**Table 1:** Profile of antiretroviral drug use in the state of Paraná and its distribution per lines of therapy and number of users

Antiretroviral drugs	Regimens including the drug	Users per line of therapy				Total users of the drug

		First	Second	Third	Not defined	
NRTI						
Lamivudine	179	21,376	10,107	2,717	67	34,267
Abacavir	42	284	286	24	7	601
Zidovudine	69	2,844	2,697	132	9	5,682
Didanosine	1	1				1
Stavudine	1		2			2
Tenofovir	101	18,415	7,117	2,547	57	28,136
NNRTI						
Efavirenz	41	14,195	39	117	1	14,352
Etravirine	37			224	1	225
Nevirapine	19	941	6	10		957
PI						
Atazanavir	55		9,404	172		9,576
Darunavir	91		1,042	2,720		3,762
Fosamprenavir	0					0
Indinavir	0					0
Lopinavir + Ritonavir	14		142	3		145
Ritonavir	143		10,186	2,884	68	13,138
Saquinavir	0					0
Tipranavir	6		1	7		8
FI						
Enfuvirtide	7			10		10
CCR5						
Maraviroc	27			67	1	68
INI						
Raltegravir	29	319	3	63		385
Dolutegravir	74	5,903	463	835	8	7,209

Results expressed as n.

NRTI: nucleoside analog reverse-transcriptase inhibitor; NNRTI: non-nucleoside reverse-transcriptase inhibitor; Pi: protease inhibitor; FI: fusion inhibitor; CCR5: CCR5 inhibitor; INI: integrase inhibitor.

The most prevalent drugs were lamivudine, tenofovir, efavirenz and dolutegravir, usually prescribed to patients as first line therapy, followed by ritonavir, a potentiator, and atazanavir and darunavir, predominantly prescribed for second and third lines of antiretroviral therapy, respectively. Some drugs, such as enfuvirtide and maraviroc, were used exclusively in third line therapy.

**Figure 1:**
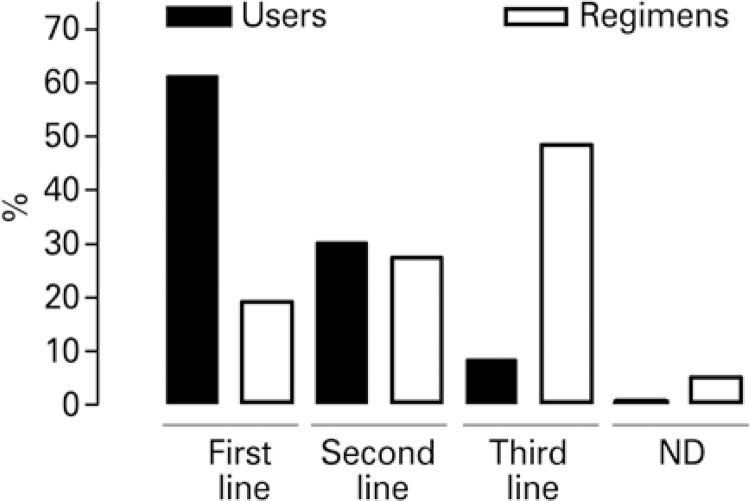
Relation among lines of antiretroviral therapy (first, second, third, and not declared) and respective number of patients on treatment in Paraná ND: not declared.

First line therapy encompassed 61.3% of users registered, for whom 19.1% of treatment regimens were prescribed. Second line therapy encompassed 27.4% of regimens identified, treating 30.2% of users. Third line corresponded to 48.5% of treatment regimens identified, which were prescribed to only 8.3% of all users registered in Paraná during the study period. The line of therapy of 5.0% of treatment regimens indicated for 0.2% of users was not defined ( Figure 1 ).

Of the total 253 treatment regimens identified, 46.2% (n=117) were not envisaged by the PCDT in effect. The regimens covered 2.4% of antiretroviral users in the state. The 64% of prescriptions not foreseen in the PCDT presented only one non-compliance, 34% had two, and 2.0% had three non-compliances.

Of the non-compliances identified, 20.5% occurred in first line treatment regimens, 29.9% in second line, and 39.3% in third line, making second line the most prevalent non-compliance.

Non-compliances identified in the antiretroviral regimens in Paraná, after comparing with the most recent PCDT, are described and quantified in table 2 .

**Table 2:** Non-compliances identified in antiretroviral therapy after comparing to the Clinical Protocol and Therapy Guidelines

Description of non-compliance found	Regimens	Users treated with regimens not included in the PCDT
Monotherapy: treatment with only one drug	7 (4.8)	161
Double therapy not allowed in the PCDT: treatment with only two drugs	35 (23.8)	207
Low efficiency triple therapy	17 (11.6)	293
Non association PI + ritonavir: mandatory association	29 (19.7)	340
Double PI	4 (2.7)	4
Triple NRTI	26 (17.0)	136
Therapeutic duplicity	4 (2.7)	11
Treatment regimens possibly guided by genotyping	25 (17.0)	50

Results expressed as (%) or n.

PCDT: Clinical Protocol and Therapy Guidelines; PI: protease inhibitor; NRTI: nucleoside analog reverse-transcriptase inhibitor.

Of total non-compliances, the most frequent regimen was double therapy not allowed by the PCDT (23.8%), followed by non-association of PI and ritonavir (19.7%), and the association of three NRTI (17.0%).

Of total individuals on regimens non-compliant with the PCDT, the non- association PI with ritonavir, low efficiency triple therapy, and double therapy not allowed in the PCDT were the scenarios in which there were more users (340, 293, and 207, respectively).

## DISCUSSION

Since the SUS made antiretrovirals available, new drugs and treatment regimens have been developed to control HIV infection. This occurs because antiretroviral therapy does not eliminate the virus from the body, and patients have to remain under treatment throughout their lives. There are many difficulties related to therapy in this scenario, such as problems with compliance, adverse reactions, drug interactions, and resistance of HIV to medication. For this reason, treatment protocols and guidelines are reviewed regularly, anticipating new antiretroviral pharmaceuticals and more effective treatment regimens, and are the major references for efficacious treatment of HIV/AIDS.^( 8 )^

The current PCDT recommends associating three antiretroviral drugs, two NRTI drugs and one of another class (NNRTI, PI/r or INI − the association of PI and ritonavir is signaled by “r”, such as atazanavir/r). The initial treatment recommended is lamivudine + tenofovir + dolutegravir, mainly when there are no related comorbidities or restrictions (for example: tuberculosis or pregnancy).^( 6 )^

In the current study, lamivudine and tenofovir, prescribed as first line HIV therapy, were the most prevalent drugs in the treatment regimens assessed in Paraná. Efavirenz, that ranks next, is associated with neuropsychiatric reactions,^( 9 )^but remains as the component of initial treatment for patients with satisfactory results and good tolerance to the drug.^( 10 )^ It is also indicated preferably as the initial regimen for child-bearing age women.^( 6 )^ Efavirenz has been gradually replaced by dolutegravir, the most recent antiretroviral added by the SUS.^( 10 )^ The change is recommended, due to higher viral suppression, less likely resistance, higher recovery of the immune system, single daily dose, and few adverse reactions attributed to dolutegravir.^( 11 - 13 )^

Ritonavir, although ranking with an expressive percentage of regimens of Paraná, should not be considered as a single drug, given it is only a pharmacokinetic potentiator.^( 14 , 15 )^

In Paraná, some drugs have stood out in second and third line therapies for HIV infection treatment. Atazanavir is a priority drug for cases of first line therapy failures, possibly because it has the advantage of being the only PI used as a daily single dose, allowing better treatment compliance.^( 6 )^ Darunavir was the most prevalent drug in third line therapy, and comprises the salvage choice after multiple treatment failures. Its indication will likely be authorized after analysis by the HIV Technical Committee.^( 10 )^

The prevalence of use of enfuvirtide and maraviroc was low in Paraná, because their use is restricted to third line therapy and genotype-guided cases. Moreover, dispensing of these drugs also depends on approval by the HIV Technical Committee of the state.^( 10 )^

As expected, first line therapy encompasses most of the population on therapy in Paraná, albeit it has the shortest list of prescribed regimens. The scenario is the contrary for third line therapy, given there are few users for many regimens prescribed. This result is expected, given first line regimens are well defined in the PCDT and prescribed for naïve patients. However, third line regimens are indicated for patients already on antiretrovirals after successive adjustments due to treatment failures, viral resistance and genotyping. Consequently, they are generally specific to each individual, impacting on the diversity of regimens prescribed and on possible non-compliances with PCDT.^( 6 )^

The classification of non-compliances identified in relation to the protocol revealed that almost half (46.2%) of antiretroviral regimens identified in the study were not included in the PCDT, but they were prescribed only for 2.26% of individuals on antiretroviral therapy in Paraná.

The PCDT does not provide all treatment options, and it is the role of prescribing professionals to indicate the most appropriate treatment whenever required, taking into account the previous use of antiretrovirals, HIV mutations (genotype resistance) and effectiveness for patients.^( 6 )^ However, some non-compliances found are strictly forbidden by the PCDT in effect, and can cause treatment failure and viral resistance.^( 6 )^ For example, monotherapy is characterized by using only one drug to treat HIV and is strongly contraindicated in the current antiretroviral therapy.^( 6 )^ Still, it was present in the treatment of 161 users in the State of Paraná. It is important to underscore that in the present study, the association PI/r was considered monotherapy. Such monotherapy may be efficacious in the suppression of viral copies, but favors the development of resistant mutations.^( 14 )^The only exception to the monotherapy included in the PCDT is to prevent mother-child transmission of HIV, recommending zidovudine as monotherapy, indicated for pregnant women during labor.^( 12 )^ However, there is no specific identification of pregnant patients on the SICLOM database. As SICLOM does not allow confirming if all monotherapies with zidovudine were prescribed to pregnant women, the indications of the drug as monotherapy were included in non-compliances, and represent a possible limitation of the study.

Double therapy was present in 26.1% of non-compliances and is not described on PCDT, because it is described as a regimen of low genetic barrier to viral resistance mutations.^( 6 )^

The PCDT suggests triple regimens for first and second lines. However, some triple regimens may be characterized as of low efficacy, since they are not effective to decrease viral load.^( 6 )^ Regimens that did not associate ritonavir and PI are not accepted by the PCDT.^( 6 )^ Ritonavir acts by inhibiting isoenzymes of cytochrome P450 3A4, decreasing the metabolism of the other associated PI, and pharmacokinetically maximizing the action of the second antiretroviral of the same class.^( 15 )^

The combination of two PI + ritonavir was not frequently prevalent in the study. However, it has been previously allowed in cases of ample resistance to PI,^( 16 )^ and currently is no longer recommended.^( 6 )^

The triple NRTI association therapy regimen is also not indicated and may lead to viral resistance.^( 6 )^ However, the triple NRTI association was indicated for 136 individuals whose treatment had some type of non-conformity. In the past, this was a treatment option for individuals coinfected with tuberculosis.^( 16 )^ Other studies have correlated triple NRTI with more likely viral mutation and consequent virus resistance.^( 17 , 18 )^

Therapeutic duplicity is characterized by double dose of the same drug at the same time, and is considered as a type of medication error^( 19 )^ related to increase in adverse reactions, without increasing efficacy.^( 20 )^

The PCDT does not define third line regimens, as they are guided by genotyping. Third line regimens can contain several classes, including the most recent, CCR5 inhibitors and IF, according to the infecting viral strain of each individual.^( 6 )^ As they are customized regimens, they are highly specific (on average two individuals per regimen). There were 17% of these regimens identified as not included in the current PCDT.

The progression of antiretroviral therapies made available by the SUS is evident. Efforts concentrated on integral care to people living with HIV in Brazil have had positive results, given access to correct antiretroviral medications, in correct dosages and with satisfactory compliance, had a positive impact on the quality of life of the population and reduced transmission of the virus.^( 21 )^

Clinical protocols guide the choice of prescribers, but they are not sufficiently comprehensive to describe the entire plurality of required treatment regimens. Regardless, indication must be careful because some choices of drugs may lead to viral resistance and treatment failure.

This study contributes to acknowledge antiretroviral drugs and regimens used to control HIV infection in Paraná. Similar studies have been conducted.^( 22 , 23 )^Considering that treatment protocols and guidelines are developed based on successful experiences of clinical studies, people living with HIV/AIDS currently on treatment with antiretroviral drugs in Paraná are estimated to have at their disposal drugs capable of providing them with treatment success, especially if allied to satisfactory compliance^( 15 )^ and appropriate outpatient follow-up.^( 24 )^The virologic success of antiretroviral therapy in Paraná was realized while the study was still ongoing, and endorses the low viral replication observed for patients on treatment.

## CONCLUSION

In the state of Paraná, during the period of the present study, treatment regimens recommended by the Clinical Protocol and Treatment Guidelines prevailed, and were prescribed for almost all patients undergoing treatment with antiretrovirals in the state. Some non-compliances identified are strictly forbidden by the Clinical Protocol and Treatment Guidelines in effect, because they may lead to treatment failure and viral resistance.

The fact that most non-compliances occurred for first and second line therapies draws attention. This is damaging to treatment success, as it increases the risk for requiring early changes in regimens to more harmful options to patients’ quality of life, and more expensive choices to the health system.
