# Supplementary material for: Antiretroviral drug use by individuals living with HIV/AIDS and compliance with the Clinical Protocol and Therapy Guidelines
Source: Einstein (Sao Paulo). 2020 Feb 13;18:eAO4995. doi: 10.31744/einstein_journal/2020AO4995 (PMC7018093; doi:10.31744/einstein_journal/2020AO4995)
Supplement: Translation

# Translation: Uso de antirretrovirais por pessoas vivendo com HIV/AIDS e sua conformidade com o Protocolo Clínico e Diretrizes Terapêuticas

**Authors:** Lucas Eduardo Fedaracz Brojan, Leticia Mara Marca, Frederico Alves Dias, Yanna Dantas Rattmann

**Affiliations:** 1 Brasil Universidade Federal do Paraná, Curitiba, PR, Brasil.

**Keywords:** Antirretrovirais, Prescrições, Diretrizes, Terapia combinada, Estudo observacional, Protocolos clínicos

## Abstract

**Objetivo:**

Descrever esquemas terapêuticos de antirretrovirais prescritos e sua conformidade com o Protocolo Clínico e Diretrizes Terapêuticas, do Ministério da Saúde, para manejo da infecção pelo HIV.

**Métodos:**

Estudo observacional e descritivo. Os dados do estado do Paraná sobre medicamentos que compõem os esquemas terapêuticos, linhas de tratamento e número de pessoas em uso destes foram acessados no Sistema de Controle Logístico de Medicamentos Antirretrovirais no período de janeiro a junho de 2018. As combinações de antirretrovirais (esquemas terapêuticos) foram comparadas conforme o Protocolo Clínico e Diretrizes Terapêuticas vigente, e as inconformidades foram categorizadas e quantificadas.

**Resultados:**

No Paraná, 35.127 pessoas com HIV foram tratadas com 253 esquemas terapêuticos distintos. Dentre os esquemas prescritos, 19,1% eram de primeira linha, 27,4% de segunda linha e 48,5% de terceira linha. Nas inconformidades, predominaram a não associação entre inibidores da protease e ritonavir (42,8%), terapia tripla de baixa eficiência (36,9%), terapia dupla (26,1%), monoterapia (20,3%) e terapia tripla de inibidores da transcriptase reversa análogos de nucleosídeos (17,1%).

**Conclusão:**

Maior parte das pessoas em tratamento do HIV no Paraná utilizam esquemas terapêuticos previstos no Protocolo Clínico e Diretrizes Terapêuticas vigente, o que contribui para o sucesso terapêutico. Entretanto, associações não previstas foram identificadas nas linhas de tratamento iniciais, podendo ocasionar inefetividade, falha virológica e resistência viral.

## INTRODUÇÃO

A infecção causada pelo vírus da imunodeficiência humana (HIV) constitui importante problema de saúde coletiva no Brasil e no mundo, em razão da morbimortalidade e do consequente impacto nas políticas públicas de saúde.^( 1 )^

Em 1996, foi promulgada a lei federal 9.313, a qual garantiu o acesso à terapia antirretroviral por meio do Sistema Único de Saúde (SUS).^( 2 )^Estudos demonstram que esta política pública voltada ao controle da infecção pelo HIV contribuiu significativamente para a redução da mortalidade e dos internamentos por HIV/AIDS no Brasil.^( 3 , 4 )^

Atualmente, o SUS disponibiliza 21 medicamentos para o controle da infecção pelo HIV, distribuídos em seis classes farmacológicas distintas: inibidores da transcriptase reversa análogos de nucleosídeos (ITRN) e inibidores da transcriptase reversa não análogos de nucleosídeos (ITRNN), que atuam impedindo a replicação do RNA viral dentro das células TCD4+; inibidores da protease (IP), que agem impedindo a enzima que fragmenta as proteínas virais sintetizadas na célula hospedeira; inibidores da integrase (INI), que atuam inibindo a enzima que integra o RNA viral no DNA da célula hospedeira; inibidor de fusão (IF), que impede a fusão da membrana viral com a membrana celular humana; e inibidor da CCR5, que inibe a proteína de membrana, que se liga ao HIV e não permite a infecção na célula hospedeira.^( 5 )^

O Protocolo Clínico e Diretrizes Terapêuticas (PCDT) do Ministério da Saúde para manejo da infecção pelo HIV norteia a escolha de prescrições por meio de esquemas terapêuticos, constituídos por combinações de mais de um fármaco antirretroviral, dispostos em diferentes linhas de tratamento. O sucesso terapêutico alcançado pelos esquemas terapêuticos proporciona a redução do número de cópias virais, o aumento do número de linfócitos TCD4+ e a consequente restauração da imunidade.^( 6 )^

A primeira linha de tratamento consiste no esquema terapêutico prescrito logo após o diagnóstico. Caso não haja sucesso na supressão viral e restauração da imunidade, a segunda linha deve ser prescrita, e assim por diante. As falhas no tratamento podem ocorrer devido às reações adversas aos medicamentos, esquemas pouco efetivos, má adesão e resistência viral transmitida. Nestas circunstâncias, os esquemas de antirretrovirais são modificados, podendo, em situações mais críticas, resultar em esquemas personalizados, guiados por genotipagem, não previstos no PCDT.^( 6 , 7 )^

Assim, torna-se importante identificar os fármacos em uso e suas associações, bem como a conformidade com o PCDT, de forma a contribuir com o uso racional dos antirretrovirais e o controle da infecção.

## OBJETIVO

Descrever os fármacos e esquemas terapêuticos antirretrovirais prescritos e sua conformidade com o Protocolo Clínico e Diretrizes Terapêuticas.

## MÉTODOS

Trata-se de estudo observacional e descritivo. Os dados secundários, referentes ao período de janeiro a junho de 2018, foram acessados via Sistema de Controle Logístico de Medicamentos Antirretrovirais (SICLOM) por intermédio do Centro de Medicamentos do Paraná (CEMEPAR), da Secretaria de Estado da Saúde do Paraná (SESA-PR).

Foram obtidos dados sobre as dispensações de antirretrovirais no estado do Paraná, estratificados por esquemas terapêuticos (combinação de diferentes antirretrovirais) dispensados, por linhas de tratamento a que pertencem estes esquemas e pelo número de usuários.

As informações foram tabuladas e, posteriormente, classificadas de acordo com a conformidade ou não em relação ao PCDT do Ministério da Saúde para manejo da infecção pelo HIV (PCDT 2017). As inconformidades foram identificadas e estratificadas nas seguintes categorias: monoterapia, terapia dupla não permitida no PCDT vigente, terapia tripla de baixa eficiência, não associação IP + ritonavir, duplo IP, três ITRN, duplicidade de tratamento e esquemas terapêuticos possivelmente guiados por genotipagem.

Esta pesquisa foi aprovada pelo Comitê de Ética da Universidade Federal do Paraná e da Secretaria de Estado da Saúde do Paraná (SESA-PR) sob o número CAAE: 82936318.3.3001.5225 e parecer: 2.674.606.

## RESULTADOS

No período de janeiro a junho de 2018, foram identificados 235 esquemas terapêuticos distintos prescritos para 35.127 pessoas vivendo com HIV/AIDS no Paraná. Dos 21 fármacos antirretrovirais padronizados pelo SUS, 18 deles foram prescritos ao menos uma vez ( Tabela 1 ).

**Tabela 1:** Perfil do uso dos fármacos antirretrovirais no estado do Paraná e sua distribuição por linhas de tratamento e número de usuários

		Usuários por linha de tratamento				
Fármacos antirretrovirais	Esquemas que o fármaco compõe					Total de usuários do fármaco
		Primeira	Segunda	Terceira	Não definida	
ITRN						
Lamivudina	179	21.376	10.107	2.717	67	34.267
Abacavir	42	284	286	24	7	601
Zidovudina	69	2.844	2.697	132	9	5.682
Didanosina	1	1				1
Estavudina	1		2			2
Tenofovir	101	18.415	7.117	2.547	57	28.136
ITRNN						
Efavirenz	41	14.195	39	117	1	14.352
Etravirina	37			224	1	225
Nevirapina	19	941	6	10		957
IP						
Atazanavir	55		9.404	172		9.576
Darunavir	91		1.042	2.720		3.762
Fosamprenavir	0					0
Indinavir	0					0
Lopinavir + ritonavir	14		142	3		145
Ritonavir	143		10.186	2.884	68	13.138
Saquinavir	0					0
Tipranavir	6		1	7		8
IF						
Enfuvirtida	7			10		10
CCR5						
Maraviroque	27			67	1	68
INI						
Raltegravir	29	319	3	63		385
Dolutegravir	74	5.903	463	835	8	7.209

Resultados expressos por n.

ITRN: inibidor da transcriptase reversa análogo de nucleosídeos; ITRNN: inibidor da transcriptase reversa não análogo de nucleosídeos; IP: inibidor da protease; IF: inibidor de fusão; CCR5: inibidor da CCR5; INI: inibidor da integrase.

Os fármacos mais prevalentes foram lamivudina, tenofovir, efavirenz e dolutegravir, comumente prescritos para pacientes em primeira linha de tratamento, seguidos pelo ritonavir, um potencializador, e atazanavir e dorunavir, predominantemente prescritos para a segunda e terceira linhas da terapia antirretroviral, respectivamente. Alguns fármacos, como enfuvirtida e maraviroque, foram exclusivamente utilizados na terceira linha de tratamento.

A primeira linha de tratamento abrangeu 61,3% dos usuários cadastrados, para os quais foram prescritos 19,1% dos esquemas terapêuticos. A segunda linha de tratamento abrangeu 27,4% dos esquemas identificados, que trataram 30,2% dos usuários. A terceira linha respondeu por 48,5% dos esquemas terapêuticos identificados, os quais foram prescritos para apenas 8,3% de todos os usuários cadastrados no Paraná no período deste estudo. Não foi definida a linha de tratamento de 5,0% dos esquemas terapêuticos, os quais foram indicados para 0,2% dos usuários ( Figura 1 ).

**Figura 1:** Relação entre as linhas de tratamento antirretroviral (primeira, segunda, terceira e não declarada) e respectivo número de pacientes em tratamento no Paraná ND: não declarada.

Do total de 253 esquemas terapêuticos identificados, 46,2% (n=117) não eram contemplados no PCDT vigente. Estes esquemas tiveram alcance de 2,4% de usuários de antirretrovirais no estado. Os 64% destas prescrições não previstas no PCDT apresentaram apenas uma inconformidade, 34% apresentaram duas e 2,0% apresentaram três inconformidades.

Das inconformidades identificadas, 20,5% ocorreram nos esquemas de primeira linha de tratamento, 29,9% na segunda linha e 39,3% na terceira linha, configurando a segunda linha como a mais prevalente.

As inconformidades identificadas nos esquemas de antirretrovirais do Paraná, após comparação com o PCDT mais recente, estão descritas e quantificadas na tabela 2 .

**Tabela 2:** Inconformidades identificadas nas terapias com antirretrovirais após comparação com o Protocolo Clínico e Diretrizes Terapêuticas

Descrição da inconformidade encontrada	Esquemas	Usuários tratados com esquemas não previstos no PCDT
Monoterapia: tratamento com apenas um fármaco	7 (4,8)	161
Terapia dupla não permitida em PCDT: tratamento com apenas dois fármacos	35 (23,8)	207
Terapia tripla de baixa eficiência	17 (11,6)	293
Não associação IP + ritonavir: associação obrigatória	29 (19,7)	340
Duplo IP	4 (2,7)	4
Triplo ITRN	26 (17,0)	136
Duplicidade terapêutica	4 (2,7)	11
Esquemas terapêuticos possivelmente guiados por genotipagem	25 (17,0)	50

Resultados expressos por n (%) ou n.

PCDT: Protocolo Clínico e Diretrizes Terapêuticas; IP: inibidor da protease; ITRN: inibidor da transcriptase reversa análogo de nucleosídeos.

Do total de inconformidades, a mais frequente nos esquemas é a terapia dupla não permitida em PCDT (23,8%), seguida da não associação IP com o ritonavir (19,7%) e da associação com três ITRN (17,0%).

Do total de indivíduos em uso de esquemas em desacordo com a PCDT, a não associação do IP com o ritonavir, a terapia tripla de baixa eficiência e a terapia dupla não permitida em PCDT foram as condições com mais usuários (340, 293 e 207, respectivamente).

## DISCUSSÃO

Desde a disponibilização de antirretrovirais pelo SUS, novos fármacos e esquemas terapêuticos foram desenvolvidos para o controle da infecção pelo HIV. Isto ocorre porque a terapia antirretroviral não elimina o vírus do organismo, e os pacientes devem permanecer em tratamento ao longo da vida. Neste contexto, existem muitas dificuldades relacionadas à terapia, como problemas na adesão, reações adversas, interações medicamentosas, e resistência do HIV à medicação. Por esta razão, os protocolos e diretrizes terapêuticas são periodicamente revisados, prevendo novos fármacos antirretrovirais e esquemas terapêuticos mais efetivos, tornando-se as maiores referências para o tratamento eficaz do HIV/AIDS.^( 8 )^

O PCDT atual recomenda associar três fármacos antirretrovirais, dois fármacos ITRN e um de outra classe (ITRNN, IP/r ou INI − a associação dos IP com o ritonavir é sinalizada pelo “r”, a exemplo do atazanavir/r). A terapia inicial recomendada é lamivudina + tenofovir + dolutegravir, sobretudo quando não há comorbidades ou restrições relacionadas (por exemplo: tuberculose ou gestação).^( 6 )^

No atual estudo, a lamividina e o tenofovir, prescritos para a primeira linha de tratamento do HIV, foram os fármacos mais prevalentes nos esquemas terapêuticos avaliados no Paraná. O efavirenz, que surge na sequência, é relacionado a reações neuropsiquiátricas,^( 9 )^porém permanece como componente de esquema inicial para pacientes com resultados satisfatórios e boa tolerância a este fármaco.^( 10 )^Também é indicado preferencialmente como esquema inicial para mulheres com possibilidade de engravidar.^( 6 )^O efavirenz tem sido gradativamente substituído pelo dolutegravir, o mais recente antirretroviral incorporado no SUS.^( 10 )^Esta troca é recomendada, devido à maior supressão viral, menor probabilidade de resistência, maior recuperação do sistema imune, dose única diária e poucas reações adversas atribuídas ao dolutegravir.^( 11 - 13 )^

O ritonavir, embora apareça com expressiva percentagem nos esquemas do Paraná, deve ser desconsiderado como fármaco isolado, por ser apenas um potencializador farmacocinético.^( 14 , 15 )^

No Paraná, alguns fármacos ganharam destaque em esquemas de segunda e terceira linhas de tratamento da infecção pelo HIV. O atazanavir se apresenta como fármaco prioritário para casos de falhas na primeira linha de tratamento, possivelmente por apresentar a vantagem de ser o único IP utilizado em dose única diária, permitindo melhor adesão à terapia.^( 6 )^O dorunavir foi o fármaco mais prevalente na terceira linha e compõe a escolha de resgate após múltiplas falhas de terapia. Sua indicação deve ser autorizada após análise da Câmara Técnica de HIV.^( 10 )^

As prevalências de uso da enfuvirtida e do maraviroque foi baixa no Paraná, pois seus usos são restritos à terceira linha de tratamento e em casos guiados por genotipagem. Além disso, a dispensação destes fármacos também é condicionada à aprovação da câmara técnica de HIV do estado.^( 10 )^

Como esperado, a primeira linha de tratamento abrange a maior parte da população em terapia no Paraná, porém contempla menor lista de esquemas prescritos. A situação se inverte na terceira linha de tratamento, pois existem poucos usuários para muitos esquemas prescritos. Este resultado é esperado, uma vez que os esquemas de primeira linha são bem definidos no PCDT e prescritos para pacientes sem uso prévio de antirretrovirais. Entretanto, os esquemas de terceira linha são indicados para pacientes já em uso de antirretrovirais, após sucessivos ajustes motivados por falhas na terapia, resistência viral e genotipagem. Consequentemente, costumam ser específicos para cada indivíduo, impactando na diversidade de esquemas prescritos e nas possíveis inconformidades com o PCDT.^( 6 )^

Na classificação das inconformidades identificadas em relação ao protocolo, obteve-se que praticamente metade (46,2%) dos esquemas de antirretrovirais identificados no estudo não estão previstos no PCDT, porém estes foram prescritos para somente 2,26% das pessoas em tratamento com antirretrovirais no Paraná.

O PCDT não prevê todas as opções de tratamento, cabendo ao profissional prescritor indicar o tratamento não previsto mais adequado quando necessário, considerando o histórico de usos prévios de antirretrovirais, mutações do HIV (resistência genotípica) e efetividade nos pacientes.^( 6 )^Entretanto, algumas inconformidades encontradas são expressamente proibidas pelo PCDT vigente, podendo ocasionar falha de tratamento e resistência viral.^( 6 )^Por exemplo, a monoterapia é caracterizada como a utilização de apenas um fármaco no tratamento de HIV e é fortemente contraindicada na terapia antirretroviral atual.^( 6 )^Mesmo assim, esteve presente no tratamento de 161 usuários do estado. Importante ressaltar que, neste estudo, a associação IP/r foi considerada monoterapia. Tal monoterapia pode ser eficaz na supressão das cópias virais, porém favorece o desenvolvimento de mutações de resistência.^( 14 )^A única exceção à monoterapia prevista no PCDT é para a prevenção da transmissão vertical do HIV, que recomenda o uso da zidovudina como monoterapia, indicada para gestantes em trabalho de parto.^( 12 )^Entretanto, na base de dados do SICLOM, não há identificação específica para pacientes gestantes. Por não permitir confirmar se todas as monoterapias com zidovudina foram prescritas para gestantes, as indicações deste fármaco como monoterapia foram incluídas nas inconformidades, caracterizando-se como possível limitação do estudo.

A terapia dupla esteve presente em 26,1% das inconformidades e não é indicada no PCDT, pois é descrita como esquema de baixa barreira genética para mutações de resistência viral.^( 6 )^

O PCDT sugere esquemas triplos para primeira e segunda linhas. Porém, alguns esquemas triplos podem ser caracterizados como de baixa eficácia, por não serem efetivos para diminuir a carga viral.^( 6 )^Os esquemas que não apresentavam a associação do ritonavir ao IP não são aceitos pelo PCDT.^( 6 )^O ritonavir age inibindo a isoenzimas do citocromo P450 3A4, diminuindo o metabolismo do outro fármaco IP associado, potencializando, de maneira farmacocinética, a ação do segundo antirretroviral da mesma classe.^( 15 )^

A combinação de dois IP + ritonavir foi pouco prevalente no estudo. Entretanto, já foi previamente permitida em casos de resistência ampla a IP,^( 16 )^não sendo mais recomendada na atualidade.^( 6 )^

A associação tripla de ITRN no esquema terapêutico também não é indicada e pode levar à resistência viral.^( 6 )^Entretanto, a associação tripla de ITRN foi indicada para 136 pessoas cujo tratamento apresentou alguma inconformidade. No passado, esta foi uma opção de tratamento de pessoas coinfectadas com tuberculose.^( 16 )^Outros estudos correlacionaram o triplo ITRN com maior probabilidade de mutação viral e consequente resistência do vírus.^( 17 , 18 )^

A duplicidade terapêutica é caracterizada pela utilização de dose duplicada de um mesmo fármaco ao mesmo tempo, sendo considerado um tipo de erro na medicação^( 19 )^relacionado ao aumento de reações adversas, sem aumento da eficácia.^( 20 )^

O PCDT não define esquemas de terceira linha, pois são guiados por genotipagem. Os esquemas de terceira linha podem conter várias classes, incluindo as mais recentes, inibidor da CCR5 e o IF, conforme a cepa viral infectante em cada indivíduo.^( 6 )^Por serem esquemas personalizados, apresentaram grande especificidade (em média duas pessoas por esquema). Nesta condição, foram identificados 17% dos esquemas não previsto no PCDT vigente.

A evolução das terapias antirretrovirais disponibilizadas pelo SUS é evidente. Os esforços concentrados no cuidado integral à pessoa vivendo com HIV no Brasil apresenta resultado positivo, pois o acesso ao medicamento antirretroviral correto, na dose correta e com boa adesão, impacta positivamente na qualidade de vida da população e reduz a transmissão do vírus.^( 21 )^

Os protocolos clínicos norteiam a escolha dos prescritores, porém não são amplos o suficiente para descrever toda a pluralidade de esquemas terapêuticos necessários. Apesar disto, é necessário ter cautela na indicação, pois algumas condutas na escolha medicamentosa podem acarretar em resistência viral e falha na terapia.

Este estudo contribui com o conhecimento dos fármacos antirretrovirais e esquemas em uso para controle da infecção pelo HIV no Paraná. Estudos semelhantes foram conduzidos previamente.^( 22 , 23 )^Considerando que os protocolos e diretrizes terapêuticas são elaborados a partir de estudos clínicos e experiências bem-sucedidas, estima-se que as pessoas vivendo com HIV/AIDS atualmente em tratamento com antirretrovirais no Paraná têm à disposição fármacos capazes de lhes proporcionar sucesso terapêutico, sobretudo se aliados à adesão^( 15 )^e ao bom acompanhamento ambulatorial.^( 24 )^Esta constatação tem ocorrido em estudo ainda em andamento sobre o sucesso virológico da terapia antirretroviral no Paraná e confirma a baixa replicação viral nos pacientes em tratamento.

## CONCLUSÃO

No estado do Paraná, no período considerado neste estudo, predominaram os esquemas terapêuticos previstos no Protocolo Clínico e Diretrizes Terapêuticas, os quais foram prescritos para quase todos os pacientes em tratamento com antirretrovirais no estado. Algumas inconformidades identificadas são expressamente proibidas pelo Protocolo Clínico e Diretrizes Terapêuticas vigente, pois podem ocasionar falha de tratamento e resistência viral.

Chama atenção o fato de que a maioria das inconformidades ocorre nas duas primeiras linhas de tratamento. Isto é negativo para o sucesso terapêutico, pois eleva os riscos de necessidade de troca precoce de esquemas para opções mais danosas para a qualidade de vida do paciente, bem como mais onerosas para o sistema de saúde.
